# An insight into Nd:YAG laser treatments for facial telangiectasia in Asian patients

**DOI:** 10.1111/srt.13516

**Published:** 2023-10-31

**Authors:** Giovanni Cannarozzo, Beatrice Marina Pennati, Tiziano Zingoni

**Affiliations:** ^1^ Lasers in Dermatology Unit Università Roma Tor Vergata Rome Italy; ^2^ El.En. Group, Clinical Research and Practice Dept. Calenzano Italy

To the editor

The Asian population is a unique group with various skin phototypes ranging from Fitzpatrick types III to V. Skin laser treatments are different from those for Caucasians because of genetic background and environmental factors. For example, race is an important factor in laser therapy in the response of the skin to inflammation. Indeed, these patients are at higher risk of pigmentary alterations and scarring after any treatment producing skin inflammation. Moreover, keloids are more likely to form in Asians than it does in Caucasians and photo ageing tends to develop later in life but with fewer wrinkling processes.^1^ Typically, this population is not characterized by vascular lesions, especially on the face area, but they are not 100% free from this issue. An example is given by little dilated vessels called facial telangiectasias (FT) that can be found on the skin's surface. They frequently affect the face and leg area.^2^ Size (0.1–2 mm diameter), hue (bluish to reddish), position, and pattern are all variables. FT are inherited in many patients, but they can also be brought on by other conditions like rosacea, connective tissue illnesses, liver disease, UV damage, elevated oestrogen levels, protracted steroid use, etc. For many people, they are cosmetic disfigurement as they are also challenging to cover with cosmetics.^3^ As expected, patients prefer effective procedures that cause little or no discomfort and have a low risk of side effects. Indeed, post‐inflammatory hyperpigmentation (typically temporary), or hypopigmentation (can occasionally be permanent and is usually caused by interference of the wavelength used with skin melanin in tanned skin or darker phototypes), or vesicular lesions due to the use of excessively high fluences (more common in darker skins), are the most frequent side effects. For this instance, long‐pulsed Nd:YAG lasers have been developed to achieve the deep skin penetration required to effectively treat widespread telangiectasias with minimal side effects.^4^, ^5^ Sure enough, the ability of the 1064 nm wavelength to hit the skin without interfering with epidermal melanin makes it possible to treat patients with darker skin tones, safely.^6^

In this study, a Nd:YAG laser (AGAIN family, Deka M.E.L.A., Calenzano, Italy) for vascular lesions was used. The study device was equipped with an external skin cooling system (Cryo6, Zimmer) during the treatment. Ten female Asian patients with a mean age of 29.9 (± 6.7) years, and III (40%) or IV (60%) Fitzpatrick phototypes were treated for facial telangiectasia. Differently from Cannarozzo et al.^7^where the pulse shape modulation was used, in this study a smaller spot of 2.5 mm, a single pulse duration of 6–7 ms and a fluence of 100–120 J/cm^2^ were set to obtain a more superficial treatment and involve just the capillaries avoiding the surrounding tissue. The treatment endpoint was the disappearance or linear blackening of the vessels. A 4‐point Global Aesthetic Improvement Scale (GAIS) (None‐0, Slight‐1, Mild‐2, Excellent‐3) was used to assess the improvement patient's skin tone after the treatment. The great majority of the study population reported an excellent (80%) or mild (20%) disappearance of the problem. They also showed to be particularly satisfied with the treatment result by the high scores regarding the global aesthetic improvement, with a mean value of 2.8 (± 0.4). Moreover, patients were asked to report pain sensation with a score from 0 (No Pain) to 3 (Very Painful). Overall, the treatment was well tolerated with a mean value of 1.6 (± 0.5) (Figure 1). Furthermore, the treatment can be considered side effect‐free (see Figure 2 for a patient's pre‐ and post‐treatment example) since patients reported only slight post‐treatment erythema and oedema that disappeared after a few hours.

**FIGURE 1:**
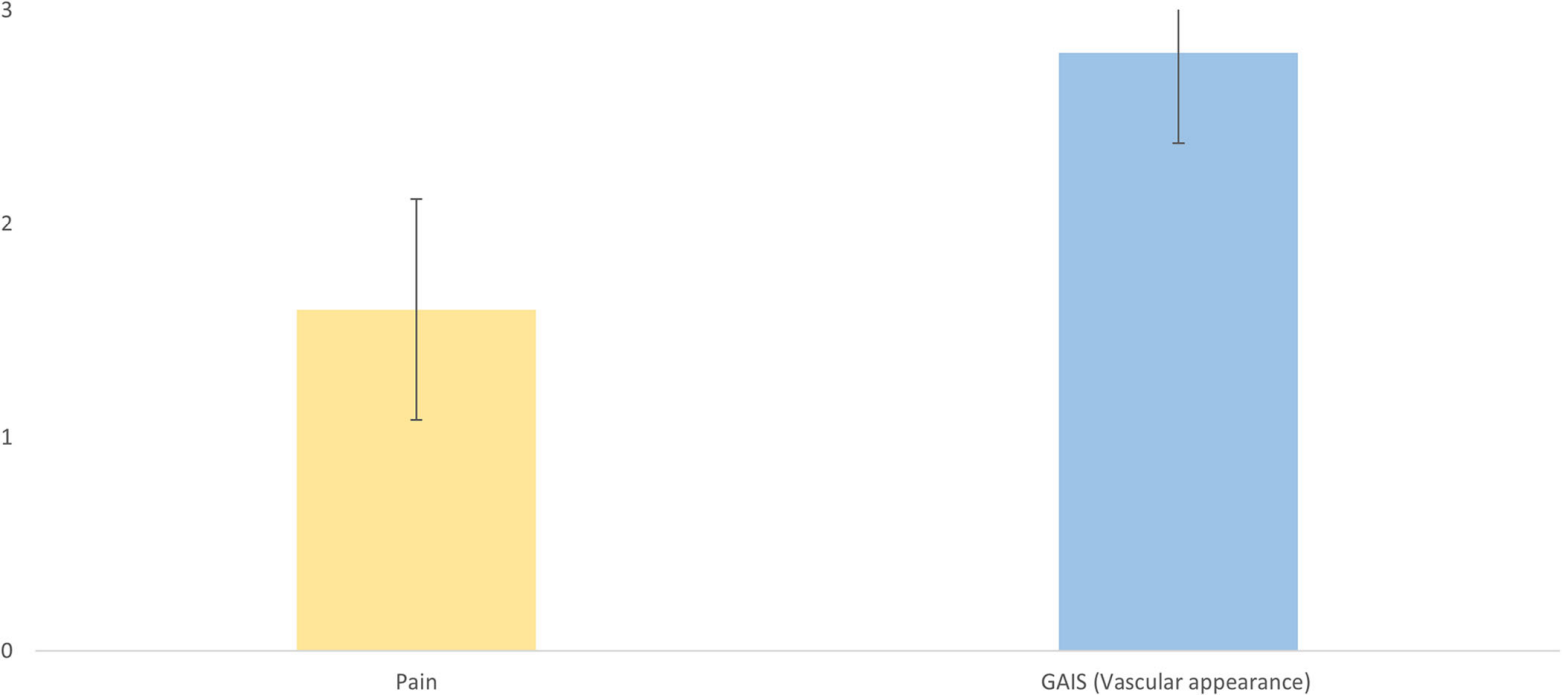
Graphical representation of the 4‐point scales used for Pain (yellow column) and skin general aesthetic (especially vascular appearance) improvement (blue column) evaluation. The patients showed a good tolerance reporting a tolerably painful treatment and high satisfaction with the final result.

**FIGURE 2:**
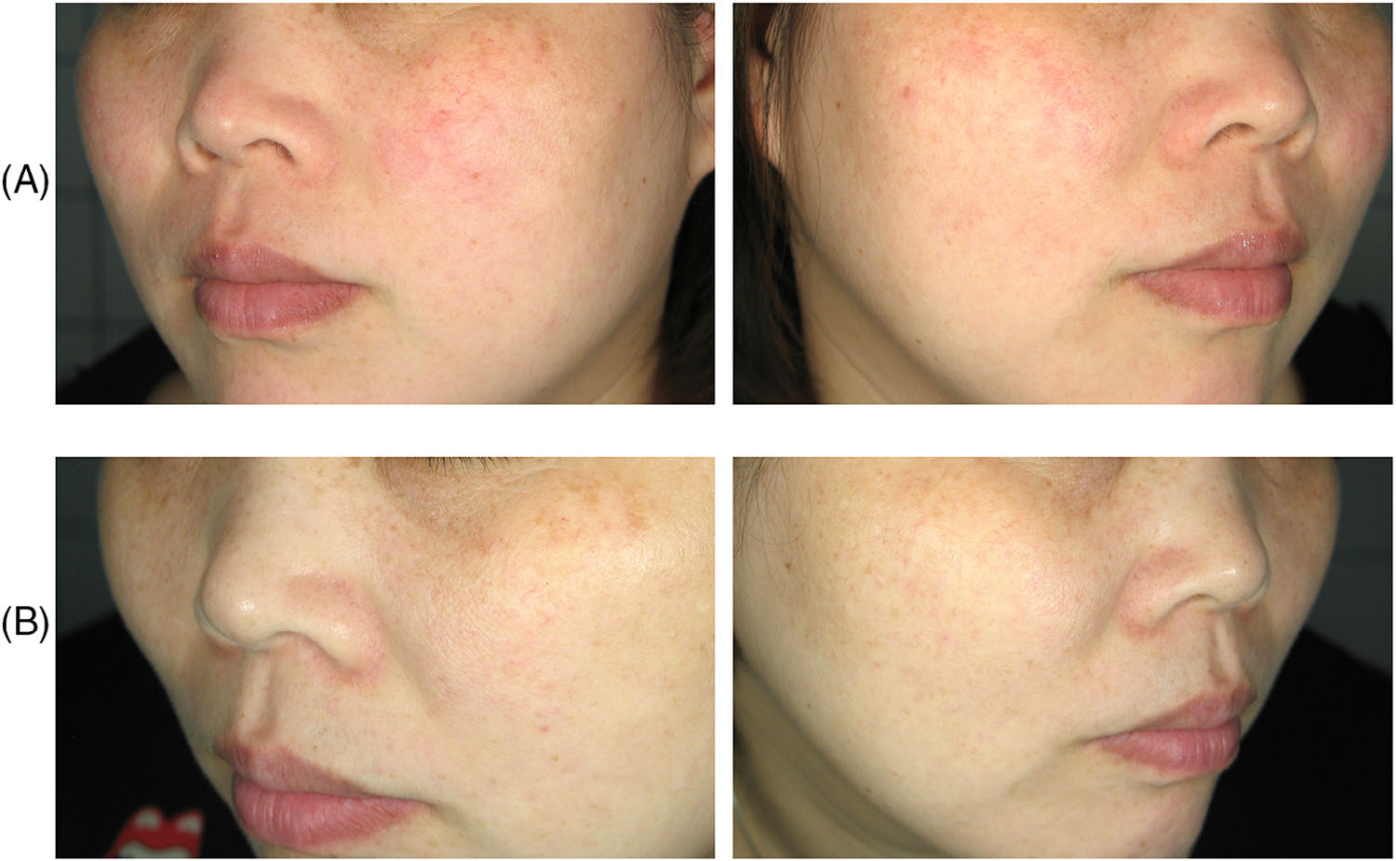
Telangiectasia treatment in an Asian woman. Pictures with polarized light were taken before (A, upper panels) and after (B, lower panels) the treatment. The patient showed no side effects.

Thanks to the post‐treatment procedures consisting of a reduction of the skin temperature with the cooling system and cold water‐wet gauze applied with slight pressure on the treated area, any possible oedema and inflammation were reduced. Thus, with this research, we further confirmed that the Nd:YAG laser can treat superficial vessels regardless of the percentage of skin melanin and so all phototypes with or without a tan are eligible^7^, ^8^ resulting in general improvement of skin vascular appearance.^9^

In conclusion, the necessity to create systems and establish treatment protocols for vascular lesions in patients with darker phototypes has been underlined by the rising demand for cosmetic solutions, pain and side effects, especially in the facial area. Pulsed dye lasers have already been demonstrated to be the most effective laser to treat vascular lesions, for example in the 10‐years’ experience of Cannarozzo et al.^10^ Clinical outcomes that were previously only possible in patients with lighter skin tones have now been made possible by technologies such as the Nd:YAG lasers. For sure, it will represent a medical leading strategy worldwide for patients with unique skin characteristics.

## CONFLICT OF INTEREST STATEMENT

B.M.P. and T.Z. are employed at El.En. Group. The authors declare that the research was conducted in the absence of any commercial or financial relationships that could be construed as a potential conflict of interest.

## Data Availability

The author has provided the required Data Availability Statement
